# Assessing biochar, clinoptilolite zeolite and zeo-char loaded nano-nitrogen for boosting growth performance and biochemical ingredients of peace lily (*Spathiphyllum Wallisii*) plant under water shortage

**DOI:** 10.1186/s12870-024-05592-6

**Published:** 2024-10-04

**Authors:** Hend Mohammad Saad Ibrahim, Abdel Wahab M. Mahmoud, Marwa Mohamed Soliman, Shaimaa Mahmoud Heider, Shady Abdel Mottaleb

**Affiliations:** 1 Agricultural Botany Department, Faculty of Agriculture, Cairo University, Giza, 12613 Egypt; 2 Plant Physiology Division, Department of Agricultural Botany, Faculty of Agriculture, Cairo University, Giza, 12613 Egypt; 3 Agriculture Research Center, Botanical Gardens Department, Horticulture Research Institute, Giza, Egypt; 4 Ornamental Horticulture Department, Faculty of Agriculture, Cairo University, Giza, 12613 Egypt

**Keywords:** Anatomy, Biochar, Biochemical components, Nano-nitrogen, *Spathiphyllum wallisii*, Zeolite

## Abstract

**Background:**

Peace lily (*Spathiphyllum wallisii* Regel) is an ornamental indoor plant with promising cut flower market, as well as antiviral, pharmacological and ecological potentials. Water deficiency can have sound effects on the growth performance and aesthetic quality of such plant. The aim of this study was to investigate the consequences of zeolite, biochar, and zeo-char loaded nano-nitrogen application on the growth performance and biochemical components of peace lily under water shortage conditions. An experiment was conducted over two consecutive seasons (2021–2022) at the experimental nursery of Ornamental Horticulture Department, Faculty of Agriculture, Cairo University, Giza, Egypt. Soil amendments; zeolite, biochar, and zeo-char loaded nano-nitrogen were prepared and applied to soil before cultivation.

**Results:**

Our results revealed that the new combination treatment (zeo-char loaded nano-N) had an exceeding significant effect on most of the studied parameters. Vegetative traits such as plant height (35.7 and 35.9%), leaf number per plant (73.3 and 52.6%), leaf area (40.2 and 36.4%), stem diameter (28.7 and 27.1%), root number (100 and 43.5%) and length (105.7 and 101.9%) per plant, and fresh weight of leaves (23.2 and 21.6%) were significantly higher than control (commercially recommended dose of NPK) with the application of zeo-char loaded nano-N during the two growing seasons, respectively. Similar significant increments were obtained for some macro- (N, P, K, Mg, Ca) and micro- (Fe, Zn, Mn) elements with the same treatment relative to control. Chlorophyll (18.4%) and total carotenoids (82.9 and 32.6%), total carbohydrates (53.3 and 37.4%), phenolics (54.4 and 86.9%), flavonoids (31.7% and 41.8%) and tannins (69.2 and 50%), in addition to the phytohormone gibberellic acid (GA_3_) followed the same trend with the application of zeo-char loaded nano-N, increasing significantly over control. Leaf histological parameters and anatomical structure were enhanced with the new combination treatment in comparison with control. Antioxidant enzymes (catalase and peroxidase), proline and abscisic acid (ABA) exhibited significant declines with zeo-char loaded nano-N treatment relative to control.

**Conclusion:**

These findings suggest that incorporating soil amendments with nano- nutrients could provide a promising approach towards improving growth performance and quality of ornamental, medicinal and aromatic species under water deficiency conditions.

## Introduction

*Spathiphyllum wallisii* Regel or peace lily, a member of Araceae, is a shade tolerant indoor ornamental plant species with little water needs [1]. Moreover, owing to its characteristic inflorescence enclosed by a large white spathe (bract), peace lily has a growing cut flower market [2]. Additionally, peace lily has 23 volatile organic compounds, mostly sesquiterpenes [3]. These volatile and aerosol constituents suggests several benefits for peace lily beside its aesthetic value. For example, it is famous for being an indoor purifier due to its ability to remove air pollutants such as benzene and toluene [1, 3]. Moreover, it could exert an “indoor forest bathing” effect, which is equivalent to the physical and psychological benefits of spending time outdoors in greenery [3]. A recent study reported that β-costol, a volatile sesquiterpene found in peace lily, could have potential antiviral properties against SARS-CoV-2 [3]. Furthermore, two groups of chlorophyll catabolites, namely: phyllolumibilins (PLuBs) and phylloleucobilins (PLeBs) were isolated from *S. wallisii*, providing good potential for use in pharmacological activities [4, 5].

Water deficiency or water shortage could affect the aesthetic value of ornamental plants and decrease their marketability due to its effect on their most wanted features; leaves and flowers [6]. Declination of leaf parameters (number, area, and color intensity) and flower characters (number and turnover) in response to water stress conditions were reported by several authors [7–14]. The reduction in growth parameters is associated with physiological and biochemical changes; some of which are adaptive mechanisms to cope with stress and improve plant performance [6]. For example, reduced leaf size and area is one of these mechanisms in which plant aims to reduce stress by decreasing surface area subjected to water loss [6]. Moreover, increment in abscisic acid (ABA) in response to water stress will stimulate stomatal closure, which in turn, will decrease stomatal conductance, photosynthetic activity and CO_2_ assimilation [6, 15, 16]. Although these mechanisms are directed towards the efficient use of available water and alleviating stress conditions, however, reduction of photosynthetic rate and CO_2_ assimilation will negatively affect the overall plant growth performance [6, 15, 16]. Decrease in photosynthetic activity is generally associated with reduction in chlorophyll biosynthesis due to alterations in thylakoid membrane structure inside the chloroplast, which are caused by water stress [17]. Additionally, the reduction in CO_2_ assimilation due to decrease of stomatal conductance will be translated into a subsequent decrement in plant’s carbohydrates content [16]. Moreover, prolonged water stress could lead to oxidative stress resulting from the excessive production of reactive oxygen species (ROS) [17]. Plant’s defense mechanism against water stress involves the production of antioxidant enzymes such as catalase and peroxidase which control and reverse damage caused by ROS [16, 17]. Non-enzymatic antioxidants such as phenols, flavonoids and tannins plays an important role in scavenging of ROS and alleviation of oxidative damage caused by water stress conditions [15, 18]. Increment in free proline content also is an important defense mechanism against stress conditions, where proline is an important osmolyte functioning to restore osmotic potential and consequently cell membrane integrity and homeostasis [17, 19]

Introducing soil amendments is one good approach for alleviating water deficiency through improvement of soil chemical and physical properties, consequently enhancing its water holding capacity and nutrient retention [17–19]. Furthermore, incorporating such amendments with nano-fertilization would offer additional benefits such as the efficient use of available resources (water and nutrients) and preserving ecosystems while improving agricultural performance [15, 17, 20]. Zeolite, a porous aluminosilicate, is an inorganic soil amendment, acting as a smart nano-carrier [20, 21]. In that perspective, zeolite provides the benefits of improved soil chemical and physical properties, increased soil water and nutrient retention for prolonged duration, provision of higher surface area for reactions and slower controlled release of nutrients, moreover, zeolite can minimize the loss of nitrogen through leaching and volatilization [20–22]. Similarly, Biochar is an organic soil amendment with characteristics similar to those of zeolite, which includes; improved soil properties and pH, high surface area, better water holding capacity, excellent adsorption and improved nutrient availability, carbon sequestration and decreased leaching [21, 23, 24]. Zeolite and biochar in addition to other soil/plant nano-supplements were reported to improve growth performance, yield and quality of many economically important plant species under water stress conditions [15, 17, 19, 25, 26].

The current research was conducted to investigate the role of zeolite, biochar separate treatments and zeo-char loaded nano-nitrogen (nano-N) complex in improving the growth performance of peace lily under water shortage conditions.

## Materials and methods

### Experimental procedure and plant material

The experiment was carried out during the 2021 and 2022 seasons at the experimental nursery of Ornamental Horticulture Department, Faculty of Agriculture, Cairo University, Giza Governorate, Egypt. Some meteorological data (monthly high, low and average temperatures, average rainfall and relative humidity) are presented in Table 1. Seedlings with 4 or 5 leaves (about 12–15 cm) of *S. wallisii* were obtained from Floramix (commercial farm in Kafr Hakim, Giza, Egypt), grown in big cork trays containing peat moss and perlite (1:1 v / v) on March 12, 2021, transplanted after 2 weeks directly in the clay loam soil, at 12–15 cm depth. Before planting, the soil was first mechanically ploughed deeply (35–45 cm) and planked twice until the soil surface had been settled. Initial soil samples were taken to identify the physical and chemical properties (Table 2) according to Jackson [27]. Farm irrigation was provided by drippers at a rate of 4 L h^−1^ as described Karmeli and Keller [28], and practiced at 7-day intervals (recommended irrigation is twice per week in clay soils and 3 times per week in sandy soils).

**Table 1:** Some meteorological parameters during the experimental period

Month	Jan	Feb	Mar	Apr	May	Jun	Jul	Aug	Sep	Oct	Nov	Dec
Min. temperature (°C)	18.9	20.5	23.5	28.1	31.7	34.0	34.6	34.4	32.8	29.4	24.3	20.3
Max. temperature (°C)	10.4	11.3	13.2	15.9	18.9	21.8	23.5	23.8	22.2	19.4	15.3	11.8
Average temperature (°C)	14.7	15.9	18.4	22.0	25.3	27.9	29.1	29.1	27.5	24.4	19.8	16.1
Average rainfall (inches)	0.10	0.17	0.20	0.08	0.00	0.00	0.00	0.00	0.00	0.08	0.18	0.15
Average relative humidity (%)	55.3	50.2	45.3	40.3	37.0	39.6	44.7	46.6	51.3	53.5	56.6	57.4

Data for average monthly min. and max. temperatures and rainfall for the nearest weather station were retrieved from weatherspark.com, while data for monthly average relative humidity were collected from weatherandclimate.com

**Table 2:** Physical and chemical properties of the experimental soil

**Physical properties**										
**Soil Texture**	**Fine Sand %**		**Coarse Sand %**		**Silt %**		**Clay %**	**Ca CO**_**3**_**%**		**O.M %**
Clay loam	43.2		4.07		16.9		35.83	4.02		1.85
**Chemical properties**										
**pH**	**EC dS/m**	**Ion concentration** **(Mmol/l)**						**Available nutrients** **(mg/kg)**		
		**CO**^**−3**^	**SO**^**−4**^	**Na**^**+**^	**Ca**^**+2**^	**Mg**^**+2**^	**K**^**+**^	**N**	**P**	**K**
7.5	1.06	0.6	3.48	1.27	6.76	7.44	3.34	68.60	11.10	120.72

### Experimental design and treatments

The experiment was carried out in a randomized complete block design (RCBD) with five replicates. During the two successive seasons of the experiment (2021 and 2022), the treatments were as follows:

- Recommended dose of chemical fertilizers (NPK) as control.
- Zeolite
- Biochar
- Zeo-char loaded nano-nitrogen

### Preparation and application of treatments

#### Application of chemical fertilizers

The recommended doses of chemical fertilizers were added. Ammonium sulphate (20.5% N) at a rate of 100 kg/feddan, calcium superphosphate (15.5% P_2_O_5_) at a rate of 150 kg/feddan, and potassium sulphate (48% K_2_O) at a rate of 50 kg/feddan were added during soil preparation. Re-application of fertilizers was divided into three doses at 40-days intervals between doses; the first was added after four weeks of transplanting.

#### Preparation and application of soil amendments and nano-materials

##### Biochar

Biochar was prepared from rice husk using the method described by Hassan et al. [29]. The husk was collected after harvesting season from El-Sharqia province, Egypt, then cut into small fragments (4–5 mm) and pyrolyzed in an oven at 350 ^*◦*^C for 24 h to produce (derive) biochar. The chemical composition of rice husk-derived biochar (BC) is presented in Table 3. The contents of ash, carbon, and hydrogen were determined according to Kinney et al. [30]. The pH in biochar weight—water volume (1:1) was determined in water suspension using a pH meter, and the EC value was measured by EC-meter. The biochar contents of Si, Ca, K, and Mg were measured by an atomic absorption spectrophotometer with air-acetylene fuel (Pye Unicam, model SP-1900, Ventura, CA, USA). Biochar was applied at the rate of 20 ton/feddan, 15 days before planting.

**Table 3:** Chemical composition of prepared rice husk-derived biochar

Property	Rice Husk-Derived Biochar
Si (mg/kg)	179
Ca (mg/kg)	213
K (mg/kg)	199
Mg (mg/kg)	179
Moisture content (%)	3.88
Ash (%)	47.90
pH	7.65
Fixed C (mg/kg)	46.35
H (mg/kg)	2.64
Sulfate (mg/kg)	0.22
Oxygen (mg/kg)	2.74
H:C	0.05
C:N	18.85
EC (dS/m)	0.14
Zeta potential (mV)	*−*26.6

##### Zeolite and zeo-char loaded nano-nitrogen

Zeolite was prepared according to Hassan and Mahmoud [31] and was added to soil at a rate of 500 kg/feddan, 10 days before planting.

**Fig. 1:**
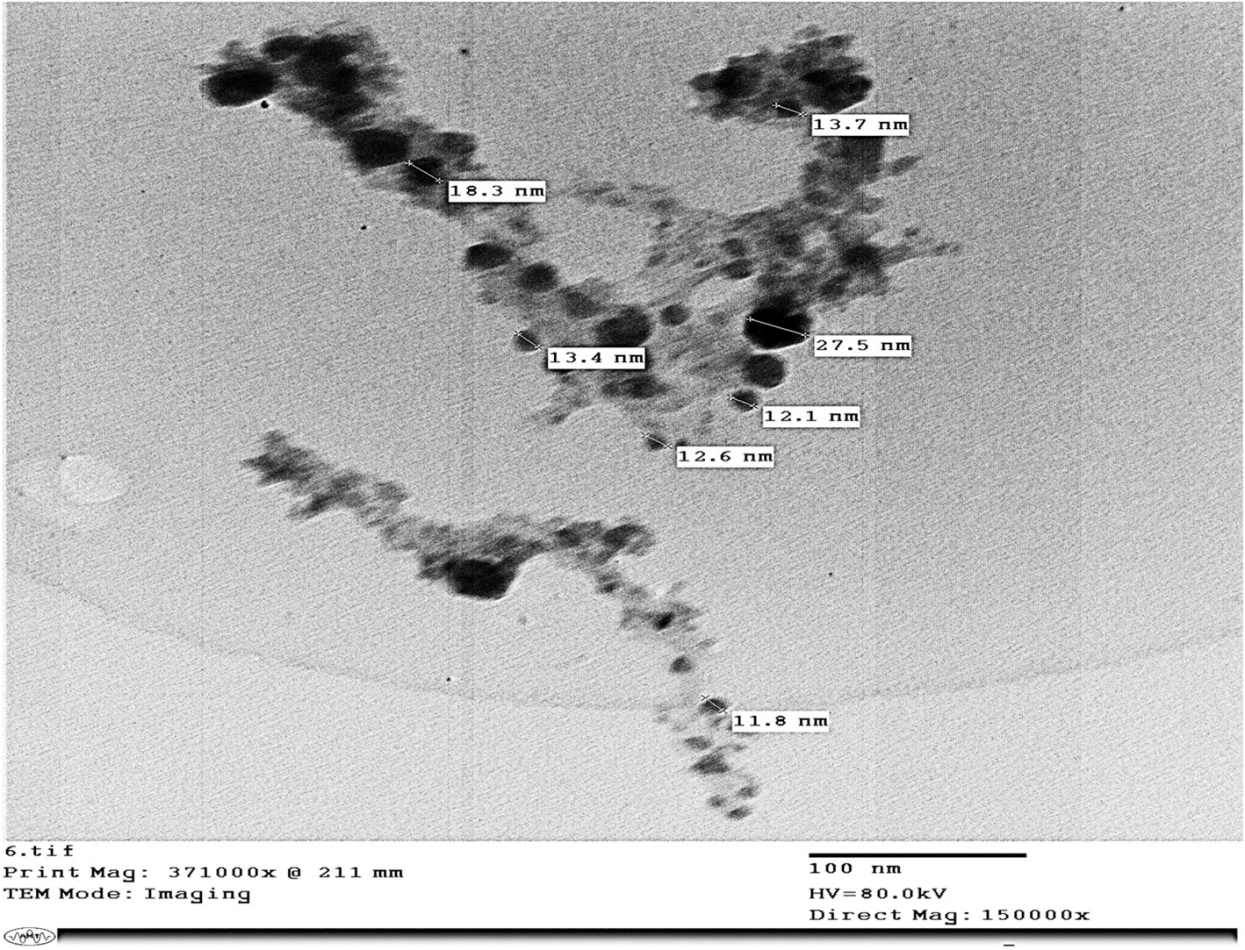
Zeolite loaded nano nitrogen examined by TEM

Prepared zeolite was loaded with nano-nitrogen (Table 4) according to Li et al. [32]. Nano nitrogen was prepared according to Mahmoud and Swaefy [18] and loaded (Fig. 1) then analyzed using the Kjeldahl digestion method [33]. Transmission electronic microscope (TEM) examination and imaging were performed at the Research Park, Faculty of Agriculture, Cairo University (FA-CURP). Zeolite loaded nano nitrogen and biochar were mixed (2:1 v:v) and incorporated into soil 12 days before planting.

**Table 4:** Chemical composition of Nano Zeolite loaded nitrogen

**Chemical Composition (%)**	**SiO**_**2**_	**TiO**_**2**_	**Al**_**2**_**O**_**3**_	**Fe**_**2**_**O**_**3**_	**FeO**	**MnO**	**MgO**	**CaO**	**Na**_**2**_**O**	**K**_**2**_**O**	**SrO**	**P**_**2**_**O**_**3**_	**N**
	45.50	2.81	13.30	5.40	8.31	0.51	6.30	9.52	2.83	0.87	0.22	0.67	2.70
**Trace elements (ppm)**	**Ba**	**Co**	**Cr**	**Se**	**Cu**	**Zn**	**Zr**	**Nb**	**Ni**	**Rb**	**Y**		
	10	1.2	35	0.8	19	64	257	13	55	15	22

### Vegetative parameters

Random peace lily plant samples were taken on 12 October 2021 as first season and 14 March 2022 as the second season for investigation and vegetative records. The following parameters were noted: plant height (cm), leaf number/ plant, leaf area (cm^2^), stem diameter (cm), root number/ plant, root length (cm), and fresh weight of leaves (g).

### Macro- and microelements

Peace lily leaves were dried and ground for preparation for chemical analysis of macro- and microelements [33]. Nitrogen (N) was determined using modified Kjeldahl method [27] while, phosphorus (P) was determined by colorimetric method (chloro-stannous molybdophosphoric blue color in sulfuric acid) described also by Jackson [27]. Flame photometer apparatus (CORNING M 410, Germany) was used to measure other macronutrients (K and Ca). Magnesium (Mg), sulfur (S) and some microelements (Fe, Mn, Zn, and Cu) were analyzed by atomic absorption spectrophotometer with air-acetylene as fuel gas (PyeUnicam, model SP-1900, US).

### Biochemical constituents

#### Photosynthetic pigments

Total chlorophyll and carotenoids were extracted from fresh leaves of peace lily plants using N, N-di-methylformamide [34]. Extracts were measured using spectrophotometer and pigments content was calculated by Moran’s formula [34], expressed in mg g^−1^ FW.

#### Total carbohydrates

Dried leaves of peace lily were used for the determination of total carbohydrates by the phosphomolybdic acid method [33].

#### Total phenolic compounds

Determination of total phenols was carried out in peace lily leaf extracts using the Folin-Ciocalteu colorimetric method described by Singleton and Rossi [35].

#### Total flavonoids

The method described by Meda et al. [36] was used for the determination of total flavonoids content in peace lily leaf extracts.

#### Tannins

Folin-Ciocalteu reagent method was used for the determination of tannins content [37] in peace lily leaves.

### Microscopic structure

Histology of leaf was studied to clarify the changes in anatomical structure between the water deficient control plants fertilized with NPK only and water deficient-zeo-char loaded nano-N treated plants. Leaf specimens were taken from the median section of the leaf blade corresponding to the median internode of both control and treated plants aged 120 days. Leaf sections were killed and fixed in formaldehyde aceto-alcohol (FAA) solution, dehydrated in butyl alcohol series, embedded in paraffin wax, sectioned transversally using a rotary microtome to a thickness of 20 µ, double stained in crystal violet-erythrosine, and finally mounted in Canada balsam as described by Nassar and El-Sahhar [38]. Photomicrographs were shot by Leica light image analysis system DM 750 for the leaf transverse sections and used to calculate the different histological measurements.

### Phytohormones, antioxidant enzymes and osmolytes

Phytohormones (gibberellic acid (GA_3_) and abscisic acid (ABA)) were determined in methanol extract of freeze-dried leaf samples according to Fales et al. [39]. GA_3_ and ABA contents were quantified and peaks were identified by ATI Unicam Gas–Liquid Chromatography, 610 Series, equipped with a flame ionization detector [40]. External authentic hormones and a Microsoft program were used to calculate the concentrations of the identified peaks. Phytohormones contents were expressed as µg g^−1^ FW of leaves.

Catalase activity was determined according to the method of Aebi [41] while, peroxidase activity was measured based on the methods of Pütter [42] and Malick and Singh [43]. Enzyme activities were expressed as units mg^−1^ protein.

Free proline was extracted from dried leaves according to Bates et al. [44] and analyzed using spectrophotometer. Content was determined as µg g^−1^ FW of leaves.

### Statistical analysis

Recorded data for vegetative and biochemical parameters as well as those for phytohormones, antioxidant enzymes and proline for water-deficient plants subjected to different treatments were analyzed using one-way analysis of variance (ANOVA) test at 5% significance level using SPSS 28.0 statistical software. Based on ANOVA results, values for different analyzed parameters were expressed as means in tables and charts (prepared by Microsoft Excel 2013) and the least significant difference (LSD) to compare different treatments was calculated using Duncan Multiple Range Test at a 5% significance level [45].

## Results

### Vegetative parameters

Mean values of vegetative parameters of peace lily exhibited significant differences (*p* ≤ 0.05) among different treatments under water deficiency conditions (Table 5). Zeo-char loaded nano-N treatment showed the highest significant values for all growth parameters in the first and second seasons, respectively in comparison with control (NPK addition only). In that concern, respective increments in both seasons were recorded in plant height (35.7 and 35.9%), stem diameter (28.7 and 27.1%), number of leaves/plant (73.3 and 52.6%), leaf area (40.2 and 36.4%), leaves fresh weight (23.2 and 21.6%), number (100 and 43.5%) and length (105.7 and 101.9%) of roots for that treatment as compared to control (Table 5). It is worthy to note that, mean values of the aforementioned characters were significantly high for either zeolite or biochar treatments separately in comparison with control, except for stem diameter for both separate treatments in first season, leaf number/ plant in both seasons, and stem diameter and root number in the second season for zeolite treatment.

**Table 5:** Effect of NPK, Zeolite, Biochar, and Zeo-char loaded nano-N on vegetative growth parameters of peace lily plants under water deficiency conditions

Character	Plant height (cm)		Leaf area (cm^2^)		Number of leaves/plant		Stem girth (cm)		No. of roots/plant		Root length (cm)		Leaves fresh weight (g)	
**Treatment**	S1	S2	S1	S2	S1	S2	S1	S2	S1	S2	S1	S2	S1	S2
**Chemical fertilizers NPK**	28.00^c^	30.67^d^	110.61^c^	114.03^c^	10.00^c^	12.67^c^	0.94^b^	0.96^c^	11.00^d^	15.33^b^	20.17^d^	22.67^d^	66.40^c^	68.37^c^
**Zeolite**	32.00^b^	34.33^c^	138.07^b^	137.50^b^	12.33^bc^	13.67^bc^	0.96^b^	1.00^bc^	14.00^c^	16.33^b^	30.87^c^	33.90^c^	74.30^b^	76.34^b^
**Biochar**	34.00^b^	37.27^b^	142.07^b^	140.93^b^	14.00^b^	16.00^b^	1.04^b^	1.03^b^	16.67^b^	20.33^a^	39.20^b^	40.57^b^	76.97^b^	78.97^b^
**Zeo-char loaded nano-N**	38.00^a^	41.67^a^	155.03^a^	155.53^a^	17.33^a^	19.33^a^	1.21^a^	1.22^a^	22.00^a^	22.00^a^	41.50^a^	45.77^a^	81.83^a^	83.12^a^
**L.S.D**_**0.05**_	**3.27**	**2.77**	**6.6**	**5.04**	**3.00**	**2.58**	**0.11**	**0.05**	**2.51**	**1.79**	**1.67**	**2.57**	**4.83**	**3.42**

S1: Season 1, S2: Season 2, Means with same letters are not significantly different at 0.05% significance level

### Macro- and micronutrients

Significant differences (*p* ≤ 0.05) in leaf macro- and microelements content were clear among all treatments of peace lily plants under water deficit (Tables 6 and 7). For macronutrients, the highest significant increments were recorded in N % (98.3 and 68.7%), P % (56 and 35.7%), K % (44.2 and 46.7%), Mg % (52.9 and 61.2%) and Ca % (57.5 and 5.3%) in both seasons, respectively, for plants treated with zeo-char loaded nano-N in comparison with control plants (Table 6). For S %, biochar and zeo-char loaded nano-N treatments scored the highest mean values in the first season, being insignificant with each other while significant with control (40.9%, each). On the other hand, S % was the highest in the second season in plants treated with zeolite, also insignificant with zeo-char loaded nano-N treatment while both being significant with control (45.5 and 40.9% for zeolite and zeo-char nano-N treatments, respectively).

**Table 6:** Effect of NPK, Zeolite, Biochar, and Zeo-char loaded nano-N on macronutrients content of peace lily plant leaves under water deficiency conditions

Character	N %		P %		K %		Mg %		Ca %		S %	
**Treatment**	S1	S2	S1	S2	S1	S2	S1	S2	S1	S2	S1	S2
**Chemical fertilizers NPK**	2.30^c^	2.75^c^	0.25^c^	0.28^c^	1.54^c^	1.54^d^	1.70^d^	1.65^c^	1.67^d^	1.32^b^	0.22^b^	0.22^c^
**Zeolite**	2.41^c^	2.51^c^	0.30^bc^	0.33^b^	1.77^b^	1.77^c^	1.84^c^	1.92^b^	1.88^c^	1.32^b^	0.23^b^	0.32^a^
**Biochar**	2.94^b^	3.52^b^	0.34^ab^	0.32^b^	1.91^b^	2.00^b^	2.02^b^	2.07^b^	2.05^b^	1.40^a^	0.31^a^	0.28^b^
**Zeo-char loaded nano-N**	4.56^a^	4.64^a^	0.39^a^	0.38^a^	2.22^a^	2.26^a^	2.60^a^	2.66^a^	2.63^a^	1.39^a^	0.31^a^	0.31^ab^
**L.S.D**_**0.05**_	**0.39**	**0.3**	**0.05**	**0.04**	**0.15**	**0.17**	**0.13**	**0.19**	**0.03**	**0.06**	**0.05**	**0.03**

S1: Season 1, S2: Season 2, Means with same letters are not significantly different at 0.05% significance level

**Table 7:** Effect of NPK, Zeolite, Biochar, and Zeo-char loaded nano-N on micronutrients content of peace lily plant leaves under water deficiency conditions

Character	Fe (ppm)		Zn (ppm)		Mn (ppm)		Cu (ppm)	
**Treatment**	S1	S2	S1	S2	S1	S2	S1	S2
**Chemical fertilizers NPK**	95.13^d^	92.99^c^	65.10^c^	65.82^c^	44.44^b^	43.14^c^	9.03^a^	8.43^a^
**Zeolite**	103.83^c^	113.00^b^	69.92^bc^	75.21^b^	52.13^a^	49.52^b^	10.46^a^	9.29^a^
**Biochar**	116.13^b^	112.43^b^	73.87^ab^	78.83^b^	48.05^b^	47.51^b^	9.37^a^	8.07^a^
**Zeo-char loaded nano-N**	118.91^a^	122.09^a^	81.31^a^	85.91^a^	52.13^a^	53.59^a^	10.15^a^	10.53^a^
**L.S.D**_**0.05**_	**2.12**	**5.76**	**7.64**	**4.88**	**3.92**	**5.51**	**4.73**	**4.79**

S1: Season 1, S2: Season 2, Means with same letters are not significantly different at 0.05% significance level

Changes in trace elements followed the same manner of macro-elements where, the highest significant increments in Fe (25 and 31.3%), Zn (24.9 and 30.5%), and Mn (17.3 and 24.2%) contents were recorded in both seasons, respectively, for plants treated with zeo-char nano-N versus control plants (Table 7). As for Cu content, no significant difference was noted among treatments in comparison with control.

### Biochemical constituents

Data in Table 8 shows significant changes (*p* ≤ 0.05) in biochemical components of leaves among different treatments of peace lily plant under water scarcity conditions. Zeo-char loaded nano-N treatment exhibited the highest increments in chlorophyll content (18.4%) in the second season, total carotenoids (82.9 and 32.6%), total carbohydrates (53.3 and 37.4%), total phenolics (54.4 and 86.9%), total flavonoids (31.7% and 41.8%) and tannins (69.2 and 50%) in the first and second seasons respectively, as compared to control plants.

**Table 8:** Effect of NPK, Zeolite, Biochar, and Zeo-char loaded nano-N on biochemical constituents of peace lily plant leaves under water deficiency conditions

Character	Chlorophyll (mg g^−1^ FW)		Total carotenoids (mg g^−1^ FW)		Total carbohydrates (%)		Total phenolics (mg g^−1^)		Total flavonoids (mg CE g^−1^ FW		Tannins (mg g^−1^)	
**Treatment**	S1	S2	S1	S2	S1	S2	S1	S2	S1	S2	S1	S2
**Chemical fertilizers NPK**	2.25^a^	2.50^b^	1.23^b^	1.41^b^	24.74^c^	26.49^c^	27.40^c^	23.90^c^	1.86^b^	1.65^b^	0.13^b^	0.14^b^
**Zeolite**	2.55^a^	2.42^b^	2.21^a^	1.77^a^	32.33^b^	33.26^ab^	35.59^b^	42.12^ab^	1.93^b^	1.88^b^	0.21^a^	0.20^a^
**Biochar**	2.38^a^	2.33^b^	1.58^b^	1.57^ab^	29.04^bc^	30.22^bc^	33.23^b^	38.38^b^	1.97^b^	1.79^b^	0.16^b^	0.21^a^
**Zeo-char loaded nano-N**	2.78^a^	2.96^a^	2.25^a^	1.87^a^	37.92^a^	36.39^a^	42.31^a^	44.66^a^	2.45^a^	2.34^a^	0.22^a^	0.21^a^
**L.S.D**_**0.05**_	**0.64**	**0.37**	**0.36**	**0.3**	**4.59**	**3.87**	**3.19**	**4.81**	**0.31**	**0.34**	**0.04**	**0.04**

S1: Season 1, S2: Season 2, Means with same letters are not significantly different at 0.05% significance level

### Histology of leaf

**Fig. 2:**
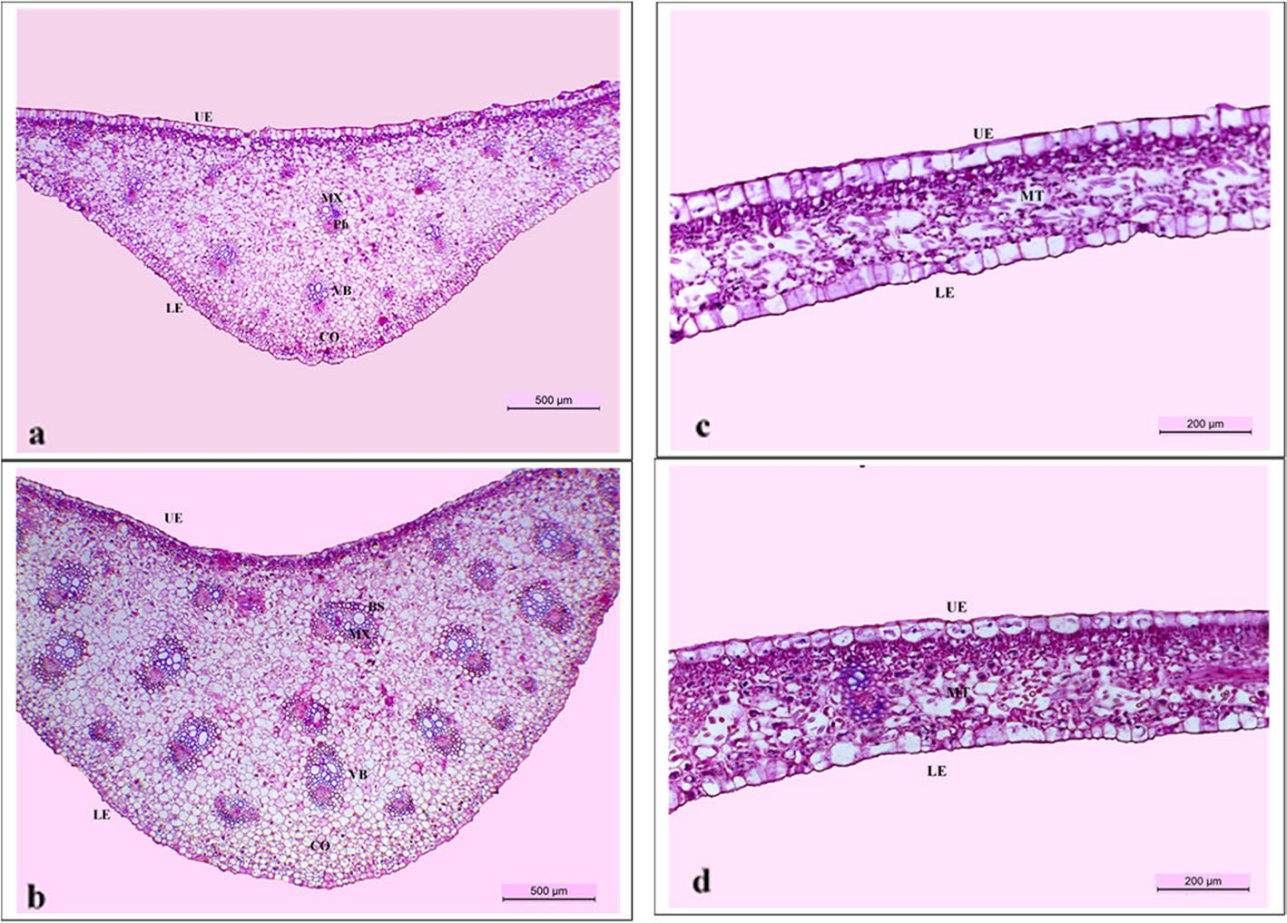
**a**, **b** Transverse sections through peace lily leaf blade, aged 120 days focusing on midrib area, a; control plants subjected to water limitation and fertilized with NPK, b; plants subjected to water limitation and treated with zeo-char loaded nano-N fertilizer. UE: upper epidermis, LE: lower epidermis, VB: vascular bundle, BS: bundle sheath, MX: metaxylem, Ph: phloem, CO: collenchyma tissue. Scale bar = 500 μm. **c**, **d** Transverse sections through peace lily leaf blade, aged 120 days focusing on laminar area, c; control plants subjected to water limitation and fertilized with NPK, d; plants subjected to water limitation and treated with zeo-char loaded nano-N fertilizer. UE: upper epidermis, LE: lower epidermis, MT: mesophyll tissue. Scale bar = 200 μm

Results regarding the anatomical structure of water deficient-NPK fertilized control and water deficient- zeo-char loaded nano-N treated plant leaves are displayed in Table 9 and Fig. 2.

**Table 9:** Histological parameters of peace lily leaf blade measured in μm, as subjected to water limitation and treated with either NPK (control) or zeo-char loaded nano-N. Results presented in table are means ± S.E, *n* = 3

Histological parameters (μm)	Water limitation + Treatments		
	**NPK (control)**	**Zeo-char loaded Nano-nitrogen**	**% ± treatment to control**
**Midrib thickness**	1281.13 ± 5.2	1821.95 ± 2.6	+ 42.2
**Lamina thickness**	279.35 ± 3.5	326.24 ± 3.9	+ 16.8
**Mesophyll thickness**	175.23 ± 1.7	228.66 ± 1.8	+ 30.5
**Number of vascular bundles (VB) in midrib**	13 ± 0.58	23 ± 1.2	+ 76.9
**Dimensions of VBs in midrib**			
**Length**	225.77 ± 6.9	330.12 ± 13.4	+ 46.2
**Width**	131.98 ± 5.9	194.89 ± 8.9	+ 47.7
**Meta-xylem vessel diameter**	35.21 ± 2.5	68.66 ± 0.54	+ 95
**Upper epidermis thickness**	50.52 ± 1.6	37.79 ± 2.3	-25.2
**Lower epidermis thickness**	45.04 ± 1.8	48.15 ± 1.9	+ 6.9

According to Table 9 and Fig. 2, increments were noted in midrib (42.2%), lamina (16.8%) and mesophyll (30.5%) thicknesses of treated plants in comparison with control plants. Moreover, increased abundance of chloroplasts was noticed in the mesophyll tissue of treated plant leaves as compared to that of control leaves. Similar increments were recorded for number (76.9%), length (46.2%) and width (47.7%) of vascular bundles, and meta-xylem vessel diameter (95%) of the midrib. On the other hand, a 25.2% decrement was found in thickness of upper epidermis in treated plant leaves versus the control one while the lower epidermis thickness increased by 6.9% in treated plant leaves over those of control plants.

### Phytohormones, antioxidant enzymes and osmolytes

**Fig. 3:**
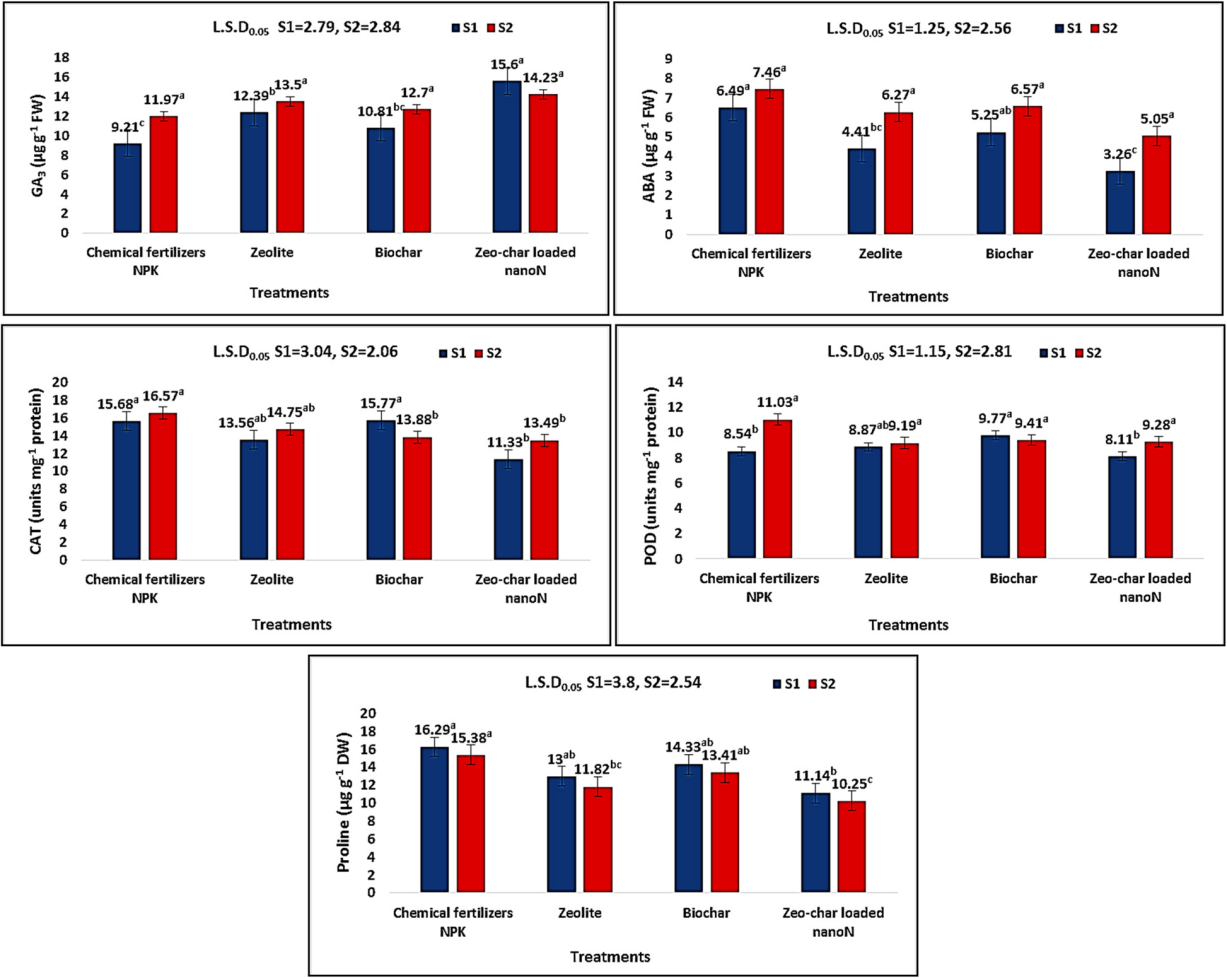
Effect of NPK, Zeolite, Biochar, and Zeo-char loaded nano-N on Phytohormones (GA_3_ and ABA), Antioxidant enzymes activity (CAT and POD), and proline content of peace lily plant leaves under water deficiency conditions, Means with same letters are not significantly different at 0.05% significance level

Mean values of phytohormones, antioxidant enzymes and proline in leaves also exhibited significant changes (*p* ≤ 0.05) in response to different treatments under water deficiency. GA_3_ increased significantly (69.4%) in the first season with zeo-char nano-N treatment while ABA decreased significantly (49.8%) with the same treatment in the same season as compared to control (Fig. 3).

In a similar manner, catalase (CAT) activity exhibited significant decrements (27.7 and 18.6%) in both seasons, respectively, with zeo-char nano-N treatment in comparison with control. While peroxidase (POD) activity in leaves being insignificantly different in case of zeo-char loaded nano-N treatment relative to control in both seasons, its activity was significantly higher (14.4%) in case of biochar treatment as compared to control in the first season (Fig. 3).

As for proline content, a significant decrease (31.6 and 33.4%) was clear in the first and second seasons, respectively, in case of zeo-char loaded nano-N treatment in comparison with control (Fig. 3).

## Discussion

*S. wallisii* or peace lily is an important indoor house plant with interestingly growing cut flower market, indoor air purification potential, and antiviral and other pharmacological properties [1–3]. Despite its little water needs [1]; water deficiency could affect the growth performance, and leaf and flower qualities of peace lily plants. On the other hand, application of soil amendments in addition to nano-N fertilization could alleviate the stress condition and improve plant growth, which will be discussed in the following lines:

### Vegetative growth parameters

Water deficit results in sound declination in growth parameters of several important ornamental species, which could drastically affect its marketability. For example, Hansen and Petersen [7] reported a 50% reduction in plant height in addition to decrements of fresh and dry weights and number of floral buds of *Hibiscus rosa-sinensis* subjected to drought conditions. Reductions in growth parameters such as plant height, leaf number and area, stem diameter, root length, fresh and dry weights of roots, shoots and leaves, and number of inflorescences of *Callistemon citrinus* were reported by Álvarez and Sánchez-Blanco [8, 10] in response to water deficit at 25% and 50% of control irrigation, respectively. Similar reductions in the previously mentioned growth parameters were recorded by Álvarez et al. [9] on zonal geranium under water stress. Elansary and Salem [11] also reported decrements in plant height, leaf number and area, root and plant dry weights of three ornamental shrubs in response to different water deficiency levels. Declination in leaf number and area, dry weights of stem, biomass and flowers of *Bougainvillea* genotypes in response to water deficiency (25% of daily evapotranspiration) was found by Cirillo et al. [12]. Similar plant growth parameters reductions were reported by other authors on several ornamental species such as *Nerium oleander* [13], *Pistacia lentiscus* [14], *Myrtus communis* [46] and *Passiflora spp.* [47] in response to water stress. In the current research, declined growth parameters for control plants of peace lily subjected to water limitation with only recommended chemical NPK supplementation matches well with the previous literature.

Reductions in growth parameters are direct results of soil water limitation on plant performance due to scarce water availability to plants, which limits water movement and hydraulic conductivity within roots, hence reducing nutrient transport –especially of N- required for plant growth, which in turn affects cell division and elongation [16, 17]. Loss of cell turgor due to declination of osmotic potential caused by water stress also has a negative impact on plant growth parameters such as root length and plant biomass [16, 19]. Other responses could be adaptive mechanisms to cope with stress consequences such as reduction in leaf size and area to reduce water loss through transpiration, hence improving water use efficiency [6, 12]. Such reduction in leaf parameters of ornamental plants could affect their aesthetic value and decrease their marketability [6, 10, 14].

On the other hand, improvements in growth parameters of peace lily was achieved with the application of either zeolite or biochar separately or in combination when both are loaded to nano-N fertilizers. Such effect is attributed to the roles of zeolite and biochar as soil amendments in enhancing physical and chemical properties of soil, hence, increasing its water retention and nutrient content. Moreover, their synergistic effect is more distinct in the combined application of zeolite loaded to nano-N and biochar (zeo-char loaded nano-N) gaining the benefit of higher water and nutrient retentions, slower and steadier nutrient release, increased reaction surface area [20, 21, 24] as well as improved nitrogen supply, which are characteristic for nano-N. Enhanced supplementation of nitrogen and other macro- and microelements is essential for enhancing cell division and enlargement, promoting plant growth [16]. Many studies were found to accord with the results of the current research. For example, Biometric parameters (stem length, diameter, and weight, leaf number, area and weight) of grass pea were enhanced with the application of zeolite under drought stress [48]. Mahmoud and Swaefy [18] found that nano-zeolite loaded N improved vegetative parameters of sage under drought conditions, while Sadowska et al. [49] reported biomass yield improvement of basil with the soil amendment with biochar and increased N fertilization rate. Same results were obtained for drought stressed barley using nano-zeolite with other soil conditioners [50]. Moreover, a combination of nano-zeolite, biochar, nano-Si and organic fertilizers was very effective in enhancing growth parameters of thyme subjected to drought-salinity combined stress [17]. Finally, the morphological traits of goldenrod were improved under water deficiency conditions with the application of a mixture of nano-zeolite loaded N and nano-Si [15].

### Macro- and micronutrients

Nutrient elements of peace lily plant were significantly lower in control plants (NPK only) as affected by water limitation. The lowest mean values of macro- (N, P, K, Ca, Mg and S) and microelements (Fe, Zn and Mn) were exhibited in this group. This could be explained by the limiting effect of water deficiency on nutrient absorption from soil and its translocation in plants due to reduced water availability to plant root system and consequent declination of root hydraulic conductivity [16, 17]. Moreover, water stress results in cell membrane damage, which drastically affects cell homeostasis and ion balance [17]. Such declination in nutrient content is very crucial to plant growth due to involvement of these elements in many essential physiological and metabolic processes such as photosynthesis, respiration, transpiration, and lipid and carbohydrate metabolism, etc. [15, 16]. Similar decrements in N, P, K, Ca and Mg were found by Hansen and Petersen [7] on *Hibiscus rosa-sinensis* affected by drought associated with low soil N and P.

In contrast, significant improvement in nutrient elements (macro and micro) content was noticed in the current study with the application of zeo-char loaded nano-N complex. Such improvement could be a result of the triple effect of biochar, zeolite, and nano-N forming up its components. As an important soil amendment, zeolite plays an important role in enhancing soil properties and water holding capacity, which improves water retention and hence increasing nutrient availability [16, 20, 21]. Moreover, zeolite is a smart nano-carrier with an excellent capacity of holding nano-fertilizers such as nitrogen, providing better retention of macro- and microelements, steadier penetration of plant tissues, and higher surface area for chemical reactions [17, 18]. In that concern, nano-zeolite loaded-N was reported to improve cation exchanging capacity, pH, and exchangeable K in soil, which enhance nutrient retention and adsorption of K, Ca, and Mg [15]. Additionally, Nano-N improves nitrogen metabolism and promotes root growth and increase its hydraulic activity, which in turn would enhance uptake of macro- and micro-nutrients from soil [15, 17, 19]. Incorporating biochar to zeolite nano-N sustains a better supply of nutrients to plants under water deficiency conditions due to its effect in improving the physio-chemical soil properties, consequently improving its water holding capacity and enhancing water and nutrient retention [21, 23]. In agreement, Improved macro- and microelements content was recorded by Mahmoud and Swaefy [18] in sage plants affected by drought conditions with the application of nano-zeolite loaded N, while improved macronutrients content in peppermint biomass with increased biochar application was obtained by Sadowska et al. [49]. Increased macro- and microelements content in drought-stressed barley was also achieved with the application of a mixture of nano-zeolite and other soil conditioners [50]. Similar results were found by Mahmoud et al. [17] on thyme in response to nano-zeolite-biochar-nano-Si mixture under combined saline-drought conditions and by Othman et al. [15] on goldenrod using a nano-zeolite-nano-Si mixture under water deficiency.

### Leaf anatomy

The histological structure of leaf blade of peace lily is composed of one-layered upper and lower epidermises with thick cuticle and numerous stomata on both. The midrib region has numerous collateral vascular bundles scattered in the ground tissue while, collenchymatous cells are found in the abaxial side of the midrib. Each vascular bundle is composed of numerous xylem and phloem elements surrounded by a sclerenchymatous sheath. The laminar area is composed of homogenous mesophyll tissue of thin-walled spongy cells abundant with chloroplasts and has large intercellular spaces in between [51].

Water deficit or limitation could induce structural changes in plant leaves, which could be direct results of such limitation, or adaptive changes to bypass such stress. For example, reduced leaf area was reported by many authors in response to water deficiency aiming at reducing water loss through transpiration on one side and photoinhibition on the other [52–55]. Reduced leaf thickness was also associated with water limitation due to decrements in mesophyll thickness. Such decrements result from decreased cell division and expansion due to shortage of water, which is important for maintenance of cell turgor and enzyme activity required for DNA replication [53, 55–57]. In the same manner, reduction in number and dimensions of vascular bundles as well as xylem vessel diameter with increased lignification was recorded in response to water stress. Such reductions could result from drought affecting procambial activity during plant growth [53], but at the same time, they are considered adaptive mechanisms to overcome such hard conditions. Narrower xylem vessels with increased lignification could maintain slower flow rate of water under stress conditions, thus reducing transpiration and preventing vessel embolism [12, 53, 57, 58]. Moreover, in response to water limitation, increased thicknesses of cuticle, upper and/or lower epidermis were explained to be adaptive mechanisms to decrease transpiration rate, protect inner tissues against damage and improve water storage capacity [55, 57, 59]. In accordance with the previous literature, this study reported reductions in midrib, laminar and mesophyll thicknesses as well as number, length and width of vascular bundles and meta-xylem vessel diameter while, thickness of upper epidermis increased in leaves of NPK-fertilized control plants subjected to water limitation in comparison with those of plants treated with a combination of zeo-char nano-N under the same limitation. Such treated plants exhibited higher mean values than control plants in all the aforementioned characters except for upper epidermis thickness, which decreased.

Improvements in leaf structural measurements could be attributed to the synergistic effect of biochar and zeolite loaded with nano N fertilizer [17]. The combined benefits of improved soil properties and consequently, water availability and nutrient uptake, are reflected directly on improvement of plant growth parameters such as leaf area and chlorophyll content which in turn would increase photosynthetic rate and improve metabolism [15]. Improved plant growth conditions would consequently result in enhanced internal plant structure. In consistency with the current study, Mahmoud et al. [60] reported improvement in leaf anatomy of caraway, represented by increased thickness of mesophyll, chlorophyll content and bigger size of vascular bundles, when plants were fertilized with nano-zeolite loaded N in combination with other treatments. Similar increments in midrib, vascular bundle, and xylem vessel diameters of drought-stressed barley leaves were found by Mahmoud et al. [50] in response to treatment of nano-zeolite combined with nano-Si and other soil conditioners. Biochar was reported to increase midrib and vascular bundle dimensions, and meta-xylem diameter of chicory leaves subjected to Pb^+^ toxicity [61]. Increased leaf laminar and mesophyll thicknesses, and vascular bundle dimensions were also reported by Hafez et al. [62] in drought stressed barley in response to biochar treatment. Similar improvements in structure and substructure were reported for barley leaves subjected to either polluted soil [63] or ZnO nanoparticles toxicity [64].

### Biochemical components

Similar to vegetative parameters and mineral content, biochemical constituents of peace lily plants are also affected negatively by water deficit, which is confirmed by lower mean values of chlorophyll, total carotenoids, total carbohydrates, total phenolics, total flavonoids, and tannins in control plants (recommended NPK supply only). Such result suggests that chemical NPK supply only was not sufficient to alleviate water deficiency conditions, probably due to loss by leaching and reduced water availability, limiting nutrient uptake. In that context, chlorophyll content is affected drastically by water deficiency and limitation of nutrient availability to plants, especially N, which is essential for biosynthesis of chlorophyll [16, 17]. Moreover, water stress causes impaired damage to thylakoid membrane in chloroplasts limiting chlorophyll biosynthesis [17]. Moreover, accumulation of ROS in chloroplasts will activate chlorophyllase enzyme activity leading to chlorophyll degradation [19, 58]. Similar declination in chlorophyll content was reported in several studies on ornamental plants subjected to water stress [13, 46, 65–67]. Following the same manner of chlorophyll, total carotenoids are reduced in response to water deficit [17]. Furthermore, total carbohydrates content is reduced due to the impairment of photosynthetic activity and decreased CO_2_ assimilation, together with depletion of available carbohydrates, which result from the effect of water stress on N availability, being an essential element in plant metabolism [16]. Additionally, reduced N availability affects biosynthesis and activity of enzymes involved in carbohydrates metabolism; increasing the activity of hydrolases (hydrolysis enzymes) and decreasing that of synthases (synthesis enzymes) [16]. Phenolic compounds, flavonoids and tannins are important secondary metabolites, which have a favorable defense role against water stress owing to their non-enzymatic antioxidant activity, directly acting in scavenging ROS and preventing oxidative stress [15, 17, 19]. Biosynthesis of phenolic compounds and flavonoids is stimulated, usually, by increase in soluble sugars resulting from carbohydrate degradation due to activity of Rubisco carboxylase under water stress conditions [15, 58]. However, reduction in these parameters was noted in control treatment as compared to other treatments, indicating a failure in counteracting water deficiency conditions. Similar to the current research, lower values of phenolic compounds, flavonoids and tannins were noted in sage [18], purslane [68], thyme [17] and goldenrod [15].

On the other hand, the highest significant values for the aforementioned contents were noted with the application of zeo-char loaded nano-N, which provides a combined synergistic effect of its three components. As previously mentioned, the complex effect of zeolite, biochar and Nano-N provides the benefits of improved soil properties and decreased leaching, better water retention, and improved nutrient uptake, majorly for N [17, 18, 20, 21, 23]. Improved pigment content (chlorophyll and carotenoids) in response to treatment implies tolerance to water deficiency and repaired photosynthetic apparatus [17, 50]. This is mainly attributed to the role of soil amendments and nano-fertilizer in improvement of soil adsorption and root system growth and nutrient absorption, which improves chlorophyll biosynthesis and accumulation of photosynthetic pigments [17, 18]. Enhanced photosynthetic rate would result in acceleration of CO_2_ accumulation, which is reflected on increased carbohydrate content [16, 18, 19, 60]. Moreover, carbohydrates metabolism is enhanced by improved nutrient uptake induced by application of soil amendments (zeolite and biochar) and nano-N [60]. Increase in carbohydrate content is associated with increments in phenolics, flavonoids and tannins, which, as previously mentioned, exert a favorable effect in plant defense mechanism, scavenging ROS and counteracting stress conditions [15, 17, 19]. Similar to this study, improvements in biochemical characters was reported by Mahmoud and Swaefy [18] on drought stressed sage under nano-zeolite loaded N treatment, Mahmoud et al. [50] on drought-stressed barley with the application of nano-zeolite and other soil conditioners, Mahmoud et al. [17] on water-saline stressed thyme using nano-zeolite-biochar-nano-Si complex, Othman et al. [15] on water deficient-goldenrod affected by the application of nano-zeolite and nano-Si mixture, and Mahmoud et al. [19] on water-stressed coriander in response to nano-zeolite application.

### Phytohormones, antioxidant enzymes and proline

Activity of enzymatic antioxidants (CAT and POD), ABA and proline contents are raised in peace lily plants in response to water deficit; NPK supply only was not able to improve stress status. While increased CAT and POD activities are important tolerance mechanisms to alleviate oxidative stress by ROS scavenging [15, 16, 69], increased proline acts to adjust osmotic pressure and restore cell turgor under water deficit conditions [17, 19]. Moreover, production of ABA in roots increase in water stress conditions and is transferred to leaves to enhance stomatal closure in order to reduce transpiration and preserve water content necessary for keeping photosynthetic function [6, 16, 17]. On the other hand, GA_3_ content is reduced under water deficit conditions due to their negative effect on cell membrane permeability and hormonal balance [16]. GA_3_ and ABA are believed to have an antagonistic interaction under stress conditions; where increase in ABA levels as an adaptive mechanism to overcome stress conditions is opposed by decrements in GA_3_ levels [17]. Many authors reported increments in antioxidant enzyme activity and proline content on ornamental plants in response to water stress [13, 65–67].

Application of zeo-char loaded nano-N complex could improve soil water content; hence, facilitate water and nutrient uptake and translocation, and consequently improve plant growth, which would alleviate water stress effect [15, 17, 19]. This would result in decrements in antioxidant enzymes activity, ABA and proline contents, and increments in GA_3_ levels as the cell regains its integrity and homeostasis and reverse the oxidative damage effect [16, 19]. Similar to the findings of this research, decrements of POD and ABA, and increments of GA_3_ were reported by Mahmoud and Swaefy [18] on water-stressed sage in response to nano-zeolite loaded N treatment. Similar decreases in CAT and ABA and increase in GA_3_ were found by Mahmoud et al. [50] in barley subjected to drought and treated with nano-zeolite and other soil conditioners. Mahmoud et al. [17] reported decrements of CAT, POD, ABA, and proline as well as increment of GA_3_ using a complex of nano-zeolite, biochar and nano-Si on water-saline stressed thyme. CAT and ABA were reduced with application of nano-zeolite loaded N and nano-Si combination to water-deficient goldenrod [15] and nano-zeolite to drought-stressed coriander [19].

## Conclusion

Peace lily is a shade-tolerant indoor plant with growing importance as a cut flower, as well as air purification, antiviral and promising pharmachological potentials. Water deficiency majorly affects growth performance and quality of peace lily plants in an adverse manner. In that concern, control plants subjected to water deficit with the application of inorganic NPK showed significant lower values for all vegetative and anatomical parameters, macro- (N, P, K, Ca, Mg, S) and micronutrients (Fe, Zn, Mn) contents, biochemical components (total chlorophyll, total carotenoids, total carbohydrates, total phenolic compounds, total flavonoids, and tannins) and GA_3_. These results indicated that the application of inorganic chemical fertilizers only was not sufficient to alleviate the negative effects of water stress on the growth performance and biochemical constituents of peace lily plant. Some parameters such as antioxidant enzymes (CAT and POD), ABA, and proline were significantly higher in case of control plants; these act usually as adaptive tolerance mechanisms against water stress. Although the separate application of biochar and zeolite as soil amendments had positive effects on growth parameters and biochemical contents of peace lily plants under water deficiency, the application of zeo-char nano-N complex had the most exceeding favorable effect on the aforementioned parameters. In brief, the complex application exerted significant increases in biometric and anatomical parameters, mineral and biochemical contents, and GA_3_, which are indicators of enhanced growth performance under water deficiency conditions. On the other hand, significant decreases were notable for antioxidant enzymes, ABA and proline; such decrements indicate that plants were able to regain cell turgor and homeostasis and counteract stress conditions. The used complex combines the benefits of zeolite and biochar as soil amendments, and nano-N fertilization, which include; improving soil properties, water availability, nutrient uptake together with their controlled release over longer duration. Improvements in growth performance and plant quality of peace lily under water deficiency conditions in response to soil amendments and nano-fertilization combination provides an eco-friendly potential as well as an efficient resource use approach for the production of such an important ornamental plant. Moreover, the introduction of zeo-char nano-N complex could provide a cost and resource effective, as well as environmentally safe alternative for chemical fertilizers, which could encourage the agricultural expansion of economically important crops in arid and semi-arid lands subjected to water shortage conditions.

## Data Availability

Data sharing is not applicable to this article.
